# Identifying patients’ support needs following critical illness: a scoping review of the qualitative literature

**DOI:** 10.1186/s13054-019-2441-6

**Published:** 2019-05-24

**Authors:** J. King, B. O’Neill, P. Ramsay, M. A. Linden, A. Darweish Medniuk, J. Outtrim, B. Blackwood

**Affiliations:** 1 Faculty of Health Sciences, School of Rehabilitation Sciences, University of Ottawa, Ottawa, Canada; 2 Centre for Health and Rehabilitation Technologies, INHR, Ulster University, Newtownabbey, Northern Ireland, UK; 3 School of Health and Social Care, Edinburgh Napier University, Edinburgh, Scotland, UK; 4 School of Nursing and Midwifery, Queen’s University Belfast, Belfast, Northern Ireland, UK; 5 Department of Anaesthesia, Southmead Hospital, North Bristol NHS Trust, Bristol, England, UK; 6 Division of Anaesthesia, Department of Medicine, University of Cambridge, Cambridge, England, UK; 7 Wellcome-Wolfson Institute for Experimental Medicine, School of Medicine, Dentistry and Biomedical Sciences, Queen’s University Belfast, 97 Lisburn Road, Belfast, BT9 7BL Northern Ireland, UK

**Keywords:** Critical illness, Qualitative research, Recovery, Scoping review, Support needs

## Abstract

**Background:**

Intensive care survivors suffer chronic and potentially life-changing physical, psychosocial and cognitive sequelae, and supporting recovery is an international priority. As survivors’ transition from the intensive care unit to home, their support needs develop and change.

**Methods:**

In this scoping review, we categorised patients’ support needs using House’s Social Support Needs framework (informational, emotional, instrumental, appraisal) and mapped these against the Timing it Right framework reflecting the patient’s transition from intensive care (event/diagnosis) to ward (stabilisation/preparation) and discharge home (implementation/adaptation). We searched electronic databases from 2000 to 2017 for qualitative research studies reporting adult critical care survivors’ experiences of care. Two reviewers independently screened, extracted and coded data. Data were analysed using a thematic framework approach.

**Results:**

From 3035 references, we included 32 studies involving 702 patients. Studies were conducted in UK and Europe (*n* = 17, 53%), Canada and the USA (*n* = 6, 19%), Australasia (*n* = 6, 19%), Hong Kong (*n* = 1, 3%), Jordan (*n* = 1, 3%) and multi-country (*n* = 1, 3%). Across the recovery trajectory, informational, emotional, instrumental, appraisal and spiritual support needs were evident, and the nature and intensity of need differed when mapped against the Timing it Right framework.

Informational needs changed from needing basic facts about admission, to detail about progress and treatments and coping with long-term sequelae. The nature of emotional needs changed from needing to cope with confusion, anxiety and comfort, to a need for security and family presence, coping with flashbacks, and needing counselling and community support. Early instrumental needs ranged from managing sleep, fatigue, pain and needing nursing care and transitioned to needing physical and cognitive ability support, strength training and personal hygiene; and at home, regaining independence, strength and return to work. Appraisal needs related to obtaining feedback on progress, and after discharge, needing reassurance from others who had been through the ICU experience.

**Conclusions:**

This review is the first to identify the change in social support needs among intensive care survivors as they transition from intensive care to the home environment. An understanding of needs at different transition periods would help inform health service provision and support for survivors.

**Electronic supplementary material:**

The online version of this article (10.1186/s13054-019-2441-6) contains supplementary material, which is available to authorized users.

## Background

The numbers of patients both admitted to and surviving intensive care (ICU) is increasing worldwide [1]. The physical, psychosocial and cognitive sequelae of critical illness, recently termed ‘Post Intensive Care Syndrome’ [2], is increasingly reported in the literature in terms of the chronicity and the impact on important patient-reported outcomes such as health-related quality of life [3], family life [4], social participation [5] and return to work [6]. This work has led to a growing international awareness of the need to support patients throughout recovery [7–9] towards survivorship [10–12]. For the purpose of this review, ‘support needs’ is defined as the additional help some adults need in order that they can live in the best way they can, despite any illness or disability they might have. They can be either short or long term, or can simply refer to the help required in getting through a difficult period.

Patients’ support needs, are not routinely assessed or addressed during patients’ ICU or acute hospital stay, and currently there are few evidence-based strategies for the translation of this increasing awareness into clinical practice [13]. Existing needs assessment questionnaires focus on a narrow or specific phase of ICU survivorship and there is limited evidence of their clinimetric or psychometric validity [14–18]. The issues are undoubtedly complex; nonetheless, a tool that could both capture patient need throughout the continuum of recovery and provide a mechanism for targeted support would be useful for the development or redesign of interventions, services or strategies.

Support needs assessment tools have been successfully developed for patient and carer populations for conditions such as cancer, traumatic brain injury and lung disease [19–21]. There are no available support needs assessment tools specifically designed for ICU survivors. In recent years, qualitative and mixed method approaches to exploring critical illness experiences has provided much needed insight into the recovery support needs from the perspectives of patients and family members. This paper describes the findings from a scoping review designed as a preliminary process towards developing such a tool for ICU survivors.

### Conceptual framework

**Fig. 1:**
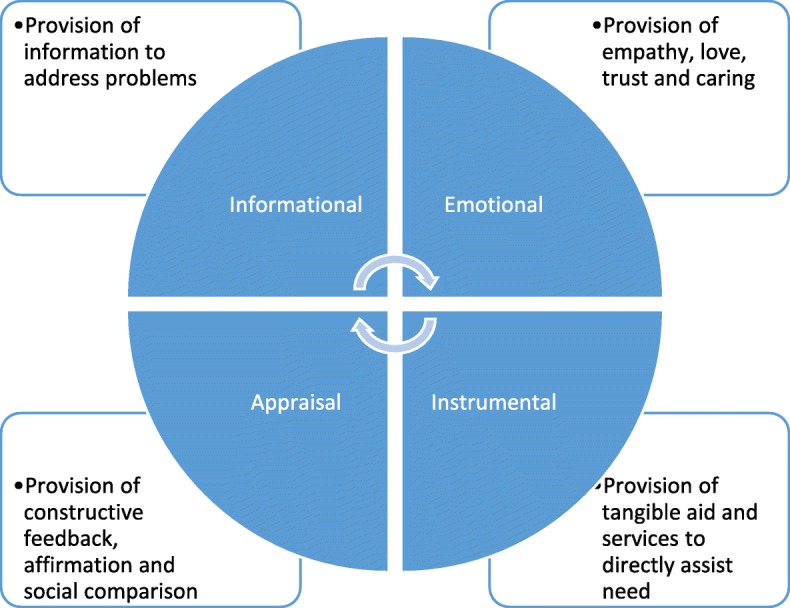
Social Support Needs framework

**Fig. 2:**
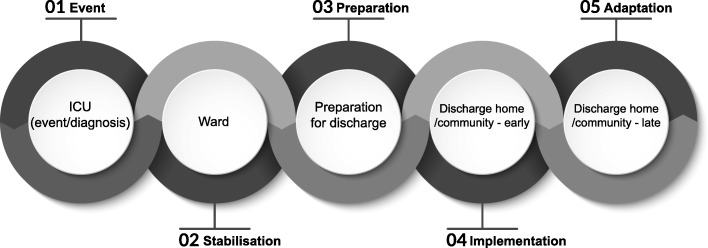
Timing it Right framework

In this review, we used the Social Support Needs framework developed by House [22] to distinguish and categorise needs into four types of support (informational, emotional, instrumental, appraisal) as shown in Fig. 1. A priori, we agreed to report additional needs if identified. To categorise corresponding support needs across the recovery continuum, we mapped the identified needs onto the Timing it Right (TIR) framework. Originally developed to capture support needs of family members caring for a stroke survivor at key recovery transition phases [23], the TIR has also been used to explore the support needs of survivors of acute respiratory distress syndrome [24, 25]. The TIR framework includes five phases of the continuum of care for ICU survivors as shown in Fig. 2.

## Methods

We developed a review protocol (Additional file 1) and reported the review according to the Preferred Reporting Items for Systematic reviews and Meta-Analyses extension for Scoping Reviews (PRISMA-ScR) [26]. We posed the following review questions: (1) what types of support do patients need following ICU discharge; (2) in what way do support needs differ across the continuum of recovery from ICU discharge to longer-term, community-based recovery?

### Search strategy

We conducted the search using key words formulated for each database [needs assessment, ICU survivorship, critical care, intensive care, qualitative research]. We searched key databases including Cumulative Index of Nursing and Allied Health Literature (CINAHL), MEDLINE, EMBASE (see Additional file 1). We limited the search from 2000 to April 2017 to capture contemporary healthcare provision.

We included qualitative research studies conducted with adult ICU patients. The phenomena of interest were patient-reported support needs that included, but were not restricted to, mental, emotional, psychological, cognitive and physical needs and resource needs such as educational and equipment needs. We included studies reporting needs at single or multiple time points after ICU discharge.

### Screening, data extraction and analysis

Two reviewers (JK, ML) independently screened titles/abstracts and full-text articles. JK and BB extracted data independently. We identified and extracted themes from eligible studies relevant to the phenomena of interest. Within the themes, we read, extracted and coded data references where authors described patient-reported needs. To ensure consistency of the coding process, data references were coded independently by two sets of three reviewers (JK and BB; JK and PR). Through discussion among the review team, we agreed that we had reached data saturation of themes and relevant codes were categorised into one of the four categories of the Social Support Needs framework and mapped against periods from the TIR framework [22, 23]. In keeping with the scoping review framework ethos, we did not apply study quality assessment [27].

## Results

**Fig. 3:**
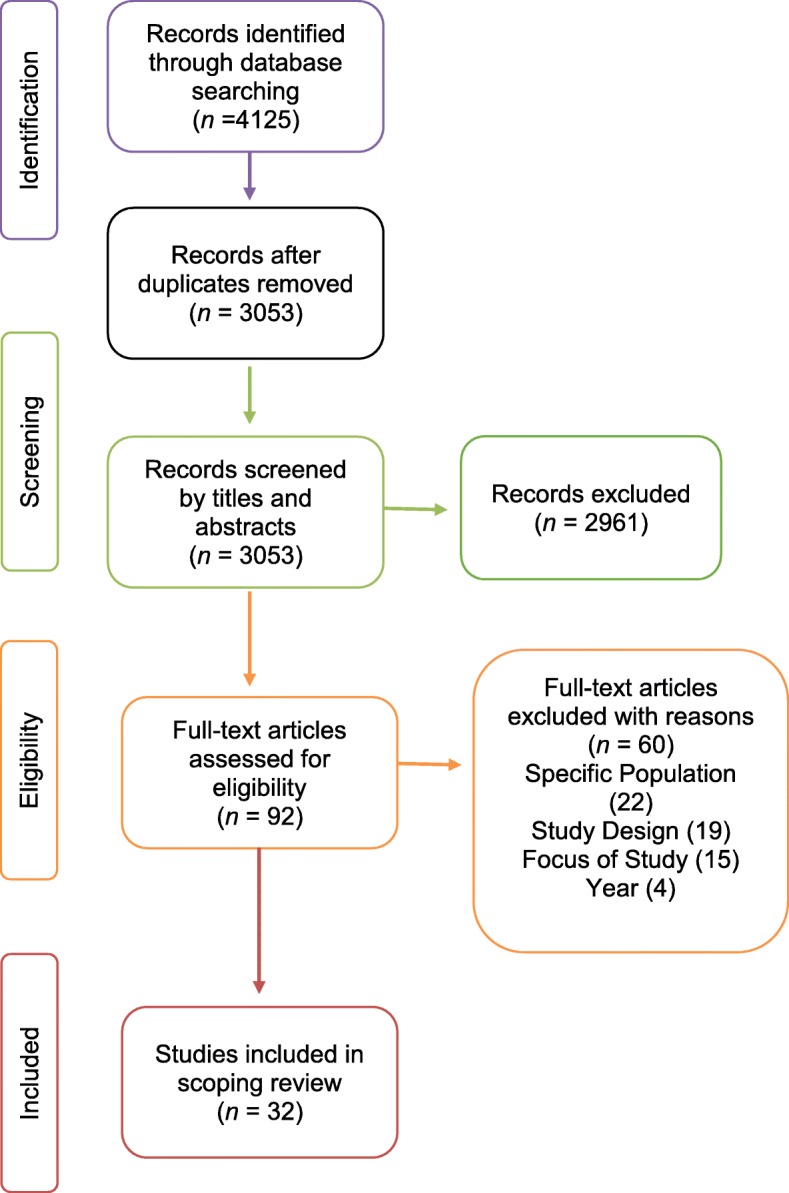
Review flow chart

We identified 3035 papers. After removing duplicates and non-eligible studies, 32 studies were included in the review (see Fig. 3). Table 1 presents the study characteristics. Study type methods included phenomenology (*n* = 6), grounded theory (*n* = 4), interpretive (*n* = 1), descriptive/narrative (*n* = 16) and survey (*n* = 4). Sample sizes ranged from five to 222, and the total number of participants in included studies was 702. Studies were conducted in the UK (*n* = 12, 38%), Australia (*n* = 5, 16%), the USA (*n* = 4, 13%), Canada (*n* = 2, 6%) and Sweden (*n* = 3, 8%); one study each (3%) conducted in Denmark, France, Jordan, Hong Kong and New Zealand, and one multinational study with participants from Australasia, Canada, the UK and the USA. Studies reported data either at single or multiple time points spanning the trajectory from ICU to post-discharge greater than 6 months (see Table 2).

**Table 1:** Included study characteristics

Study	Country	Time point focus	TIR Phase	Sample size *N* = 702	Approach and methods	Data collection timing
Abdalrahim 2014	Jordan	Hospital discharge to 3-months	Implementation	18	Descriptive Individual interviews	3 month post hospital discharge
Adamson 2004	Australia	ICU and hospitalisation	Event/diagnosis; stabilisation/preparation	6	Descriptive Individual interviews	6 month post hospital discharge
Agard 2012	Denmark	First 12 months after D/C from ICU	Stabilisation/ Preparation/implementation/Adaptation	17	Grounded theory Dyad interviews (spouse and patient) Focus group interviews	3 and 12 months post ICU discharge
Bench 2011	UK	ICU transfer to ward	Stabilisation	11	Descriptive Focus group interviews	Variable from < 3 months to 3-years
Bench 2014	UK	ICU transfer to ward	Stabilisation	42	Survey	Prior to hospital discharge
Chaboyer 2003	Australia	ICU, ward and home	Event/diagnosis; stabilisation/preparation; Implementation/adaptation	222	Descriptive Individual interview and group meetings	ICU, ward, 3, 6, 9, 12 months post hospital discharge
Chaboyer 2005	Australia	ICU transfer to ward	Stabilisation	7	Descriptive Focus group interviews	1–2 months post hospital discharge
Chahraoui 2015	France	ICU stay / current psychological state (3 months)	Event/ diagnosis stabilisation/preparation; Implementation	20	Survey/descriptive Questionnaire/individual interviews	3 months post ICU discharge
Chiang 2011	Hong Kong	ICU, ward and home	Event/diagnosis; stabilisation/preparation; Implementation/adaptation	6	Grounded theory Individual interviews	Variable, ICU, ward, and 3 months post ICU discharge
Cox 2009	USA	ICU to home	Event/diagnosis; stabilisation/preparation; Implementation/adaptation	23	Phenomenology Individual interviews	Variable, 3, 9, or 12 months post hospital discharge
Cypress 2011	USA	ICU	Event/diagnosis	5	Phenomenology Individual interviews	Ward
Czerwonka 2015	Canada	ICU, ward, home	Event/diagnosis; stabilisation/preparation; Implementation/adaptation	5	Descriptive Individual interviews	Variable, 3, 6, 12, 24 months post ICU discharge
Deacon 2012	USA, UK, Canada, Australia, NZ	ICU and Post ICU discharge	Event/stabilisation/ preparation; Implementation/adaptation	35	Survey Questionnaire	Unreported time, post hospital discharge
Field 2008	UK	ICU transfer to high dependency unit/step down or ward	Stabilisation	34	Descriptive Individual interviews	Variable, post hospital discharge
Haraldsson 2015	Sweden	2-month post ICU discharge	Implementation	12	Descriptive Individual interviews / diaries	2 month post ICU discharge
Hupcey 2000	USA	In ICU	Event/diagnosis	14	Grounded theory Individual interviews	In ICU or ward
Hupcey 2001	USA	In ICU	Event/diagnosis	30	Descriptive Individual interviews	In ICU or ward
Jones 2003	UK	ICU transfer to ward	Stabilisation	18	Descriptive Case study Individual interviews	Within 1 week of ICU discharge and 6 months post ICU discharge
Lee 2009	Canada	ICU, ward, home	Event/diagnosis; stabilisation/preparation; Implementation/adaptation	25	Descriptive Individual interviews	Approximately 6 years post ICU discharge
Lof 2008	Sweden	Falling ill,, ICU, ward	Event/diagnosis; stabilisation/preparation	9	Descriptive Individual interviews	3 and 12 months post ICU discharge
Maddox 2001	Australia	Returning home from hospital	Implementation	5	Interpretative Individual interviews	6–15 weeks post ICU discharge
Magarey 2005	Australia	ICU	Event/diagnosis	8	Survey/descriptive Questionnaire / Individual interviews	Up to 2 years post ICU discharge
McKinney 2002	UK	ICU transfer to ward	Stabilisation	6	Phenomenology Individual interviews	In ICU and in ward
Minton 2005	NZ	ICU, ward, home	Event/diagnosis; stabilisation/preparation; Implementation	6	Descriptive Individual interviews	6 months post ICU discharge
Odell 2000	UK	ICU transfer to ward	Stabilisation	6	Phenomenology Individual interviews	Ward
Palesjo 2015	Sweden	ICU, ward, home	Event/diagnosis; stabilisation/preparation; Implementation/adaptation	7	Phenomenology Individual interviews	Up to 2 years post ICU discharge
Pattison 2015	UK	ICU and ongoing recovery needs	Event/diagnosis; implementation	22	Grounded Theory Email interviews	2–4 weeks or 6 months post hospital discharge
Prinjha 2009	UK	ICU follow-up care after hospital discharge	Implementation/adaptation	34	Descriptive Individual interviews	Post hospital discharge
Ramsay 2013	UK	ICU transfer to ward	Stabilisation	20	Descriptive Individual interviews	Post hospital discharge
Ramsay 2016	UK	Post ICU discharge to hospital discharge	Preparation	14	Descriptive Focus group interviews	> 3 months post ICU discharge
Strahan 2005	UK	ICU transfer to ward	Stabilisation	10	Phenomenology Individual interviews	3–5 days on the ward
Williams 2009	UK	Illness experience/critical incident and its aftermath	Event/diagnosis; stabilisation/preparation; Implementation/adaptation	5	Blended discourse, narrative and phenomenological approaches Individual interviews	Early post hospital discharge and 1 year later

**Table 2:**
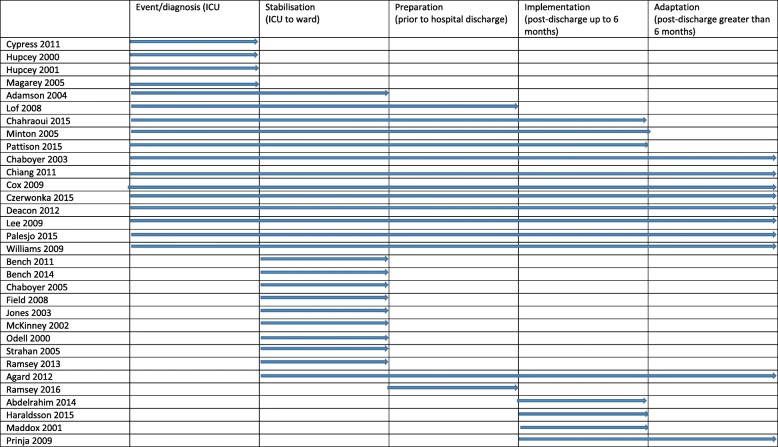
Study reported time-periods according to the Timing It Right framework

### Findings

We report findings in the four categories of support (i.e. information, emotional, instrumental, appraisal) with reference to the phases of the TIR framework.

#### Informational needs

Informational needs changed across the care continuum from event/diagnosis (ICU admission) to the adaptation phase.

#### Event/diagnosis (ICU admission)

In ICU, patients’ informational needs centred on the events surrounding the ICU admission, diagnosis, treatment and prognosis [25, 28]. Reflecting the acuity of illness accompanied by prolonged use of sedation to facilitate treatment (e.g. mechanical ventilation) and the prevalence of delirium, patients reported memory loss and a sense of being ‘drugged’ [24, 28–32]. Patients reported a need for information to enable them to understand the events surrounding their ICU admission and an understanding of their current health status, including their inability to speak and think clearly [24, 28]. Patients struggled, however, to integrate their own fragmented memories with factual information provided by ICU staff [28]. A key support need reported during this phase was for repeated transfer of clear, easily understandable information from healthcare staff to patients and families [25, 28].

#### Stabilisation (ward care)

The need for continued, clear communication was also apparent in the transition from ICU to the hospital ward. Patients reported a lack of communication between ICU and ward staff to facilitate continuity of care [33–35]. Although ICU discharge summaries were helpful, patients felt the information was too basic and needed more specific details, tailored to their unique presentation [33, 34, 36]. When information was provided, patients recalled periods of memory loss and not knowing where they were [36]. While some patients attributed this to not receiving information, others indicated that there was an element of forgetting because everything was ‘blurred’ and highlighted the need for continual repetition of information and orientation [37].

#### Preparation (ward care)

As patients progressed towards preparation for hospital discharge, their informational needs changed, to focus on progress made since ICU discharge and the treatments and medications needed to ensure ongoing recovery [25]. The reported information needs continued to focus on the illness event and prognosis, as patients began to realise the nature, severity and short and long-term implications of their critical illness [25].

#### Implementation/adaptation (discharge home)

At home, information needs continued to focus on understanding their critical illness, but with a greater emphasis on coping with the long-term sequelae and stress. Return visits to the ICU, seeing the room they had occupied, and using an ICU diary were seen as beneficial by some patients in filling in the gaps, but not for others [38]. Patients wanted information delivered in a more permanent fashion, such as pamphlets or booklets for ongoing review [25]. Patients indicated a sense of wanting more information, but not knowing where to obtain it [24, 25]. They needed information and education to be extended to family members [39], particularly as questions about their experience and medical condition persisted long after returning to the community [24]. The need for the full ‘story’ was expressed by survivors to enable them to make sense of, and reclaim ownership of, their lives [28].

### Emotional needs

#### Event/diagnosis (ICU admission)

Patients experienced a wide array of emotional reactions that changed over time. Recalled emotions prior to intubation in the ICU were terror, dread, uncertainty and facing imminent death [31, 40]. Patients described regaining consciousness after a life-threatening condition as confusing, shattering and a feeling of emptiness [40]. Initial reactions included death anxiety [5, 28, 31, 32, 40–43], feelings of loss of control [30, 37, 40], powerlessness [29], panic and abandonment [5, 44]. Fear and anxiety were common reactions to being physically restrained, endotracheal suctioning, chest physiotherapy, nasogastric tune insertion, the inability to communicate and having a tracheostomy [40, 41, 45].

Needs expressed during the early initial stages included the need for comfort [29] in words and touch [30] and the support of family [25, 28, 32, 44–47]. The need for family support and attendance extended across time. Within the ICU, knowing relatives could be contacted easily helped patients to develop a coping strategy [46] and the family support led to feelings of happiness and security [25].

#### Stabilisation (ward care)

Not surprisingly, the need that patients expressed for security and familiarity was often jeopardised when they transitioned from the familiar environment of the ICU to the new environment of the ward. Relocation anxiety was experienced by some patients when transferred to the ward [37], despite the presence of critical care outreach follow-up for some patients [35, 48]. Conversely, some patients experienced a sense of detachment, compliance and acceptance resulting in contentment: they had entered a chain of events over which they had no control [49]. Patients cited difficulty adjusting to the change from a one nurse to one patient ratio in the ICU to a lower nurse to patient ratio in the ward [49]. The ratio change caused patients to feel abandoned and vulnerable because of the loss of closer relationships with nurses [50, 51] as well as feeling unimportant [50], isolated and neglected [51]. In addition, many patients felt depressed because of a perception of poor physical progress following transfer [35, 49].

#### Implementation/adaptation (discharge home)

Patients found the first few months after hospital discharge the most difficult and felt insecure about no longer being in the safe hospital environment [52]. Following discharge, vivid memories of ICU experiences involving terrifying dreams and flashbacks [41, 42, 50], and fear and worry about the complexities of their illness persisted for months [24, 25]. During this time, patients needed a lot of reassurance. Yet, one study reported that patients were reticent about seeking telephone support from ICU follow-up clinic nurses, even though the nurses had urged them to do so, due to a presumption that they were busy or had forgotten them [52]. The lack of contact resulted in some patients feeling abandoned after hospital discharge [52], and where scheduled follow-up ICU visits were provided, patients reported these were preceded with feelings of nervousness and tension brought on by unpleasant memories [38]. Follow-up sessions provided some security in allowing opportunity to ask questions and gain knowledge of their stay in the ICU [38].

Variability in the emotional experiences of survivors was common on discharge and was influenced by the availability of support at home. Patients with no primary caregivers experienced more anxiety and fear, while those with family members and support networks were more optimistic and positive about their discharge [25]. Furthermore, other patients felt a loss of role within the family and feelings of being dissociated and not involved in family decisions [5] and helplessness [53].

#### Adaptation (discharge home)

Patients’ reported that their re-integration back into the community caused increased stress and was a source of depression [25]. Some expressed a sense of isolation as they avoided socialising, such as visiting relatives because it provoked unpleasant memories [5]. As a result, some patients expressed a need for mechanisms to allow an emotional outlet for themselves and their family members, including the support of community-based healthcare providers [25]. Across a few studies, patients felt that, unlike their physical health, their emotional and psychological health had received little attention and would have valued psychological counselling, more support from community-based healthcare providers and support in re-building psychological independence and confidence [25, 39, 46, 52]. Some patients reported they benefited from a support group where they had met others who truly understood the experience.

### Instrumental needs

#### Event/diagnosis (ICU admission)

During their time in ICU, patients reported discomfort arising from a debilitating lack of sleep, noise, fatigue, pain and anxiety [29, 31, 32, 35, 40, 41]. Key instrumental needs reported by patients were for personal care, hygiene and comfort, particularly relating to bathing, nutrition and pain relief [31, 54]. As patients moved to the ward, they reported a need to progress from dependent to independent care, but needed adequate professional support to achieve that. Chiang et al. [46] summarised patients’ views on needing structured continuity of care, such as that delivered by a critical care outreach service, and sufficient professional support before discharge home to the community. Additionally, patients in one study noted that they rarely experienced continuity of medical care [24].

#### Stabilisation (ward care)

Transferring from the ICU to the ward resulted in patients struggling to cope with basic care previously provided by nurses in the ICU [49, 51]. Some patients assumed that they had to undertake their own basic care either because ward nurses were ‘too busy’ or because communication between the ICU and ward had broken down and ward staff were unaware of the patient’s support needs [51]. Although some patients accepted they needed to be more independent on the ward, they still needed considerable physical help from either the staff [33, 35] or family carers [51].

#### Implementation/adaptation (discharge home)

A dominant theme across all TIR phases and particularly in the post ICU discharge period was the profound and disturbing physical and cognitive disability experienced by patients. For some, there were trauma-related disabilities such as loss of a limb or paralysis [41], loss of muscle strength and tone resulting in inability to stand [29], and decreased strength and endurance [24, 39]. Patients reported they struggled for independence to re-establish their premorbid physical strength [40, 55]. Lesser-reported functional issues were problems with vision, speech and hearing [53]. Substantial, persistent cognitive deficits were also reported [41, 53, 55], with a need for continued observation and support from caregivers to prevent harm due to patient forgetfulness [41]. Patients with cognitive impairment had to relearn performing basic behaviours in personal care and household activities; and at 1 year, goals shifted to higher level functioning such as planning, organisation, driving and returning to work [55].

Patients reported feelings of being a burden resulting from their lack of independence, and felt that their weakened state compromised their ability to lead a normal life [24]. Patients reported they needed physical support at home from community-based healthcare providers to assist them to become independent [25, 43]. They cited the need for earlier follow-up appointments where these were available, rather than months later [52]. Even after 1-year substantial training, many patients had not returned to their pre-ICU level of strength and activity [55].

Changes in living status due to increased reliance on support from family and friends, inadequate financial assistance and reduced family income were problems cited by some survivors [53]. Swedish patients also described the need for support from society to find appropriate work to prevent falling into financial difficulties with paying housing and hospital bills [43].

### Appraisal needs

#### Stabilisation (ward care)

Appraisal needs were not evident during the ICU stay, but following transfer from ICU, many patients noted that ward staff knew little about them and therefore could not provide feedback on how they were progressing [51]. Some patients concluded this was due to lack of communication between the ICU and the ward [51]. Others expressed positive aspects of the transfer out of ICU; feeling this indicated an improvement in their recovery [37].

Following ICU discharge, patients could appraise how far they had come, citing feelings of doing well since their ICU stay [44, 45], and feeling special to have survived critical illness [42, 45]. In the study by Jones [42] which included only males, patients were able to identify their strong points and capitalise on them, but others failed to appreciate the mental and physical transformation required and how long this took after critical illness [41, 45]. Palesjo et al. [43] described the critical illness recovery process as a time when patients struggled to return to ordinary life, strived for reconciliation and learned to live in the moment in a changed body. In some cases, patients described their visible and invisible body marks as continuous reminders of their critical condition [43] and these often resulted in family relationship strain and change [41]. Life adjustment to the changes occurring after ICU required building up defence and coping mechanisms such as active coping, positive reframing, humour, acceptance, optimism, hope, self-sufficiency, goal-setting and spirituality [41, 44].

Patients stated they benefited from meeting others who had been through the ICU experience and understood the challenges they were addressing [39, 53]. They expressed an overwhelming desire to know that what they experienced was ‘normal’, and that it took a long time and should not be concerned with slow progress [38, 39, 52]. Patients gained comfort from identifying with others’ experiences, and this helped normalise their own experiences [53].

#### Spiritual needs

An additional category of spiritual support needs emerged from the literature and was not necessarily synonymous with religious needs. Three studies reported patients’ views about having near death experiences and the need to believe in a higher entity [5, 32, 49]. A study conducted in Jordan reported survivors needing to thank and praise Allah for their recovery, making Dua (the act of supplication or asking Allah for help), and wishing to visit holy places to show obedience to Allah [5]. Similarly, Magarey and McCutcheon [32] reported that patients described a spiritual experience of moving from powerlessness to a sense of purpose and acceptance in their recovery. For some patients transferring from the ICU to the ward resulted in them realising that ‘I could have died’ [32, 49]. This traumatic realisation caused many participants to revisit the meaning of their lives and make each day count [49].

## Discussion

This review has categorised ICU survivor support needs across the ICU patient recovery trajectory and has shown how they exist, change and adapt over time. Identifying and understanding the overwhelming emotional, physical and cognitive experiences, and the subsequent support needs expressed by people who have had a stay in ICU, is a powerful step towards determining early service intervention as patients make their journey from ICU to regaining independence at home.

Our scoping review confirms that patients’ support needs are undoubtedly multifaceted and complex following critical illness. Patients express various needs at each transition point. In the early phases, instrumental and emotional needs come to the fore reflecting the fundamental human needs for nutrition, hydration, comfort, safety and physical and emotional support. Some support needs persisted and/or evolved across the continuum of recovery, depending upon the level of disability. If not addressed early on, these needs would likely continue and escalate in a later phase of recovery. As patients transitioned into different phases, their support needs followed the pattern of Maslow’s hierarchy of needs [56]: requiring safety and security as they transitioned to the ward; needing family support and belonging and needing a sense of esteem as they transitioned towards increased independence from hospital care and the cotton wool blanket of family support. Although we used House’s Social Support Needs framework to classify needs [22], we kept an open mind to capture additional needs. Spiritual needs emerged as an additional category in this population of patients which is unsurprising given the high mortality rates that have been reported in multinational cohort studies for patients during (19%) and after (24%) an ICU admission [57].

We consider the use of House’s classification of needs with the addition of spiritual needs as highly relevant to this patient population. This view is supported by a recent study exploring contributory factors to early-unplanned hospital readmission of ICU survivors and recommending that interventions and service redesign include a strong focus on social support [58]. Contributory factors were inadequate informational (communication between secondary/primary care, hospital discharge planning, medication communication), emotional and spiritual (timing of psychological care, coming to terms with near-death experiences), instrumental (mobility issues and problems with specialist aids/equipment) and appraisal (fragile social support and goal setting) needs.

This review showed that patients were sometimes able to meet their own needs by drawing on previous life experience and this provided them with an element of ‘appraisal’ not captured by House’s original definition, e.g. they showed ability to assess their own internal appraisal as opposed to receiving external appraisal from others [22]. Conceivably, ability to appraise may reflect self-efficacy or greater ability and motivation to manage their own recovery—a concept termed patient activation. While there are various methods for assessing aspects of activation, such as self-efficacy [59], health locus of control [60] and readiness to change [61], they focus on predicting single behaviours rather than the broader elements such as knowledge, skills, beliefs and motivation that a patient needs to manage a chronic illness [62].

Because support needs change at different stages of recovery, a method of identifying greatest need according to the patient’s phase of recovery may help to target specific services at appropriate times. Developing a method, tool or questionnaire that could capture individual patient needs at any stage of recovery after ICU would be useful in clinical practice as this could help target care, strategies and services to support each individual and enable optimal provision of support to meet their changing needs. Additionally, services that are not yet available could be identified and established. While there has been consideration for needs assessment and needs-driven care in other populations (e.g. cancer care, coronary artery disease, interstitial lung disease) [63–65], we believe this review could inform a needs assessment tool or questionnaire for critical care survivors.

The strengths of our review include the use of identified frameworks for categorising support needs and recovery phases. The literature on ICU survivorship is quite large; therefore, we focused our search to include qualitative studies about patients’ needs. We conducted our search only up to April 2017 and found there were repeating themes within the papers suggesting we had reached data saturation. Our assumption was confirmed by a recent study of contributory factors for readmission of ICU survivors reporting similar patient and system level themes [58].

## Conclusion

Our review is the first to identify and summarise the changes in social support needs among intensive care survivors across the continuum from intensive care to the home and community environment. Patient needs are complex after ICU and should be assessed for each individual so that needs driven care and services can be appropriately provided to help recovery. Future research could consider the results from this review if developing a needs assessment tool for the critical care population.

## Additional file

Additional file 1: Review protocol. (DOCX 24 kb)

## Abbreviations

ICU: Intensive Care Unit
TIR: Timing it Right
