# Supplementary material for: Identifying patients’ support needs following critical illness: a scoping review of the qualitative literature
Source: Crit Care. 2019 May 24;23:187. doi: 10.1186/s13054-019-2441-6 (PMC6533750; doi:10.1186/s13054-019-2441-6)
Supplement: Supplementary file 1 — Review protocol. (DOCX 24 kb) [file 13054_2019_2441_MOESM1_ESM.docx]

# Additional File 1. Scoping Review Protocol

# **Title:** Patient-reported support needs during and after the critical illness event: a protocol for a scoping review

## Authors: King J, O’Neill B, Ramsay P, Linden M.A., Darweish Medniuk, A, Outtrim J, Blackwood B.

**Methods**:

The purpose of this scoping review is twofold: (1) to explore and identify patients’ support needs and (2) to locate them within and across the dimensions and timeframes of the Timing it Right framework

The scoping review will apply the framework by Arksey and O’Malley (Arksey & O'Malley, 2005), further refined by Levac (Levac, Colquhoun, & O'Brien, 2010). Which includes 6 steps.

1. Identify the research questions
2. Identifying relevant studies
3. Selecting Studies
4. Charting the data
5. Collating, summarizing and reporting results
6. Consultation with stakeholders

**1- Research Questions**

1. What types of support do patients need following ICU discharge?
2. In what way do support needs differ across the continuum of recovery from ICU discharge to longer-term, community-based recovery

**2-Identifying relevant studies**

**Search Strategy**

The research team will meet to provide their experience and methodological expertise to further guide the identification of relevant studies. A research librarian will help guide the search working closely with the team. A specialised search strategy will be developed in consultation with the librarian.

**Data Sources**

**Databases**

Databases will be searched will include the Cochrane Library, Cumulative Index of Nursing and Allied Health Literature (CINAHL), Education Source, EMBASE, ERIC, Joanna Briggs Institute, MEDLINE, Nursing Allied Health Database, PsycINFO, and PEDro (Physiotherapy Evidence Database)

**Inclusion/Exclusion Criteria**

**Language**-We will include studies in English only.

**Date**-from 2000 to present

A decision was made to limit the searches from the year 2000 onwards. The purpose of the scoping review is to identify studies reflecting the needs of patients in ICU, and it was felt that prior to 2000 there have been significant differences in health care delivery in ICUs around the world.

**Keywords and concepts**

**Key concepts** of the review are intensive care/critical care; patient support needs; qualitative research.

**Keywords**- keywords will depend on each database but will include words such as critical illness, intensive care, critical care, needs assessment, ICU survivor, qualitative research/studies, support needs.

**Types of participants (age, condition)**

Searches will be restricted to those conducted with humans.

We will include studies of adults (18 years plus) who have experienced an ICU admission and were discharged from the ICU. We will exclude studies of family and/or carers’ needs. We will exclude studies that only focus on the specific needs of a specific patient population.

**Types of phenomena**

We will include studies whose purpose was

- To explore support needs of patients including mental, emotional, psychological, cognitive, and physical needs and other resource needs such as educational and material needs.
- To explore a patient’s needs over time (key constructs, domains, themes) with view to seeing how they change/alter over time. Either for the purpose of developing a support needs survey or to plan services or an intervention.

We will exclude papers that solely focus on one domain e.g. QOL; physical function.

**Timing of data collection in studies**

We will include studies that focus on any single time point post ICU discharge or studies that have included data collection across multiple points over time.

**Types of studies**

We will include any type of qualitative research studies, including mixed methods that include a qualitative component.

We will exclude review studies. But we will access the review study included studies to determine whether or not those studies meet the inclusion/exclusion criteria for our scoping review.

**Grey Literature**

As the focus of this scoping review is to find research studies a decision was made not to include the grey literature.

**3- Selecting Studies**

For the studies identified from each database, studies’ titles will be independently screened by two reviewers [JK and ML] against the inclusion and exclusion criteria.

Abstracts will be independently reviewed by the two reviewers [JK and ML] that appear to meet the inclusion and exclusion criteria.

Full text of studies appearing to meet inclusion and exclusion criteria will be screened by both reviewers

If there is a disagreement between JK and ML if a study should be included or excluded into the scoping review then BO’N will review the study to make the final decision on inclusion or exclusion.

**4- Charting the data**

The data from the studies will be charted using an excel sheet and appropriate tables.

**5- Collating, summarizing, and reporting results**

The results of the scoping review will be summarized against the research questions, as well by the descriptive headings of the studies including citation information, country, purpose, participants, setting, timing of data collection, qualitative research approach used.

All themes relevant to the phenomena of interest will be identified and within the themes, we will extract and code data where authors have described patient-reported needs. Data will be coded independently by reviewers, and discussed with the wider review team. Data will be categorised into one of four categories of the Social Support Needs framework (informational, emotional, instrumental, appraisal) [House 1981] and mapped against periods from the TIR framework (event/diagnosis reflecting ICU stay; stabilization/preparation reflecting the ward stay; and implementation/adaptation reflecting discharge to the community) (Cameron & Gignac, 2008) to identify corresponding support needs across the recovery continuum.

**6- Consultation with stakeholders**

The results of this review will be integrated with other key steps for developing a needs assessment questionnaire; including information from expert health care professionals and ICU survivors. The data sources will be used to inform the development of a questionnaire which can be used to assess support needs of ICU survivors at key transition stages, and throughout the recovery process.

**References**

Arksey H, O'Malley L. (2005) Scoping studies: towards a methodological framework. *International Journal of Social Research Methodology, 8*, 19-32. doi:10.1080/1364557032000119616

Cameron JI, Gignac MA. "Timing It Right": a conceptual framework for addressing the support needs of family caregivers to stroke survivors from the hospital to the home (2008) *Patient education and counseling.* 70(3): 305-14

House JS. (1981) Work stress and social support: Reading, Mass. : Addison-Wesley Pub. Co.

Levac D, Colquhoun H, O'Brien KK. (2010) Scoping studies: advancing the methodology. *Implement Sci, 5*, 69. doi:10.1186/1748-5908-5-69
